# Incidence of gout in spondyloarthritis

**DOI:** 10.1093/rap/rkag039

**Published:** 2026-03-28

**Authors:** Stuart Johnston, Benjamin Zuckerman, Aashmi Sharma, Uazman Alam, Sizheng Steven Zhao

**Affiliations:** School of Cardiovascular and Metabolic Health, University of Glasgow, Glasgow, UK; Rheumatology Physiotherapy, South Sector, NHS Greater Glasgow and Clyde, Glasgow, UK; Centre for Rheumatic Diseases, King’s College London, London, UK; Centre for Musculoskeletal Research, Division of Musculoskeletal and Dermatological Science, School of Biological Sciences, Faculty of Biological Medicine and Health, University of Manchester, Manchester Academic Health Science Centre, Manchester, UK; Department of Cardiovascular and Metabolic Medicine, Institute of Life Course and Medical Sciences, Liverpool Centre for Cardiovascular Science, University of Liverpool and Liverpool University NHS Foundation Trust, Liverpool, UK; Centre for Musculoskeletal Research, Division of Musculoskeletal and Dermatological Science, School of Biological Sciences, Faculty of Biological Medicine and Health, University of Manchester, Manchester Academic Health Science Centre, Manchester, UK; NIHR Manchester Biomedical Research Centre, Manchester University NHS Foundation Trust, Manchester, UK

Dear Editor, Psoriatic disease, including PsA and psoriasis, is closely linked with metabolic disorders such as obesity, type 2 diabetes, hyperuricaemia and gout. The strong association between PsA and gout has led researchers to coin the term ‘psout’, reflecting shared pathophysiology and metabolic comorbidities [1]. The risk of gout is elevated in psoriasis [hazard ratio (HR) 1.71 (95% CI 1.36, 2.15)] and even higher in PsA [HR 4.95 (95% CI 2.72, 9.01)] [2]. However, incidence rates (IRs) across other spondyloarthropathies remain limited to a single case–control study [3]. Gout has a prevalence of approximately 4% and is associated with adverse cardiovascular outcomes, reduced health-related quality of life and high healthcare resource usage [4, 5]. Understanding the incidence of gout across spondyloarthropathies is therefore crucial. Managing coexisting gout and SpA can be challenging, particularly in differentiating SpA flares from gout attacks. This study aimed to describe the incidence of hyperuricaemia and gout across psoriatic disease, axial SpA (axSpA) and enteropathic arthritis.

We used electronic health records from North American healthcare organisations. Details on the database are available at trinetx.com and have been previously described [6]. Study populations included individuals ≥18 years of age with at least two International Classification of Diseases, Tenth Revision (ICD-10) codes for PsA, psoriasis, enteropathic arthritis or axSpA (Supplementary Table S1). We also included RA for context and restricted to seropositive RA (ICD-10 code M05) to improve diagnostic specificity and minimise overlap with other inflammatory arthritides. Follow-up began 1 day after the first ICD code for the index disease. Outcomes included incident gout (M1A) and incident gout or hyperuricaemia (without signs of inflammatory arthritis or tophaceous disease, E79.0 or M1A). Due to significant heterogeneity in demographics, clinical features and treatments across inflammatory arthropathies, our primary aim was to describe incidence rather than conduct statistical comparisons.

We identified 44 434 individuals with axSpA [mean age 46.8 years (s.d. 17.9), 61.8% male], 72 831 with PsA [51.8 years (s.d. 15.6), 42.5% male], 92 696 with psoriasis [48.5 years (s.d. 19), 49.7% male], 2579 with enteropathic arthritis [43 years (s.d. 17.2), 34.2% male] and 33 113 with seropositive RA [59.3 years (s.d. 15.1), 23.9% male]. IRs of gout per 1000 patient-years were highest in PsA [11.8 (95% CI 11.4, 12.2)] and axSpA [11.0 (95% CI 10.5, 11.6)], comparable to RA [11.4 (95% CI 10.7, 12.1)]. Psoriasis and enteropathic arthritis had lower IRs (Fig. 1). As expected, marginally increased IRs were observed when hyperuricaemia was included in the case definition across axSpA, PsA, enteropathic arthritis, psoriasis and RA (Fig. 1).

**Figure 1:**
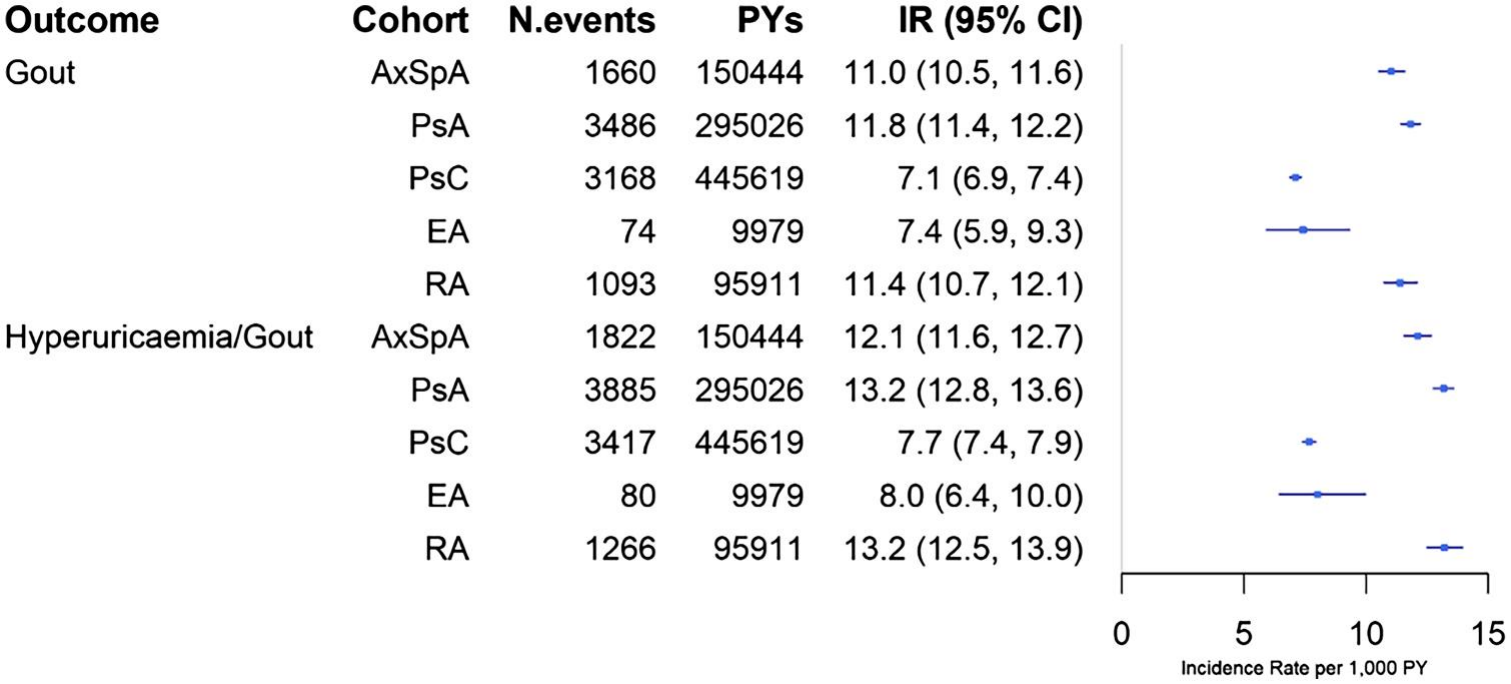
IR per 1000 patient-years (PYs). PsC: psoriasis; EA: enteropathic arthritis

These findings align with prior studies reporting a high incidence of gout and hyperuricaemia in PsA but also highlight the novel finding of similarly high incidence in axSpA. This builds on previous work reporting increased gout prevalence in axSpA compared with the general population (1.94% *vs* 0.56%, respectively) [3]. While IRs were still elevated, incidence was lower in cutaneous-only psoriasis and enteropathic arthritis. Consistent with prior studies [7], we also observed an elevated incidence in RA, although this cohort was older. Risk factors such as obesity and cardiometabolic comorbidities, well-documented in PsA and psoriasis [1–3], are increasingly recognised in axSpA [8]. This may explain the comparable IR observed for axSpA in our study. Research on enteropathic arthritis is limited and this cohort comprised the smallest population in our study. The reason for the lower incidence of gout in this group is unclear and warrants further investigation.

Clinicians should consider gout as a differential diagnosis in peripheral mono-/oligoarticular flares of SpA, not limited to PsA or RA. Individuals with SpA already face a substantial burden of multimorbidity, and these data highlight the clinical importance of gout as a comorbid condition that may impact health-related quality of life and outcomes [4, 5]. While this study utilises a large dataset, the potential for misclassification using ICD codes exists. Nevertheless, it lays the foundation for future research to identify key risk factors, including obesity, serum uric acid levels and intestinal dysbiosis [8].

In conclusion, this study demonstrates a high incidence of gout in axSpA, comparable to PsA. Clinicians should consider gout as a differential diagnosis in acute mono-/oligoarticular flares of SpA.

## Supplementary Material

rkag039_Supplementary_Data

## Data Availability

The data that support the findings of this study are available from TriNetX (trinetx.com), but third-party restrictions apply to the availability of these data. The data were used under license for this study with restrictions that do not allow for the data to be redistributed or made publicly available. However, for accredited researchers, the TriNetX data are available for licensing at TriNetX. To gain access to the data in the TriNetX research network, a request can be made to TriNetX (trinetx.com), but costs may be incurred, a data sharing agreement will be necessary and no patient identifiable information can be obtained. No data from Liverpool University Hospitals NHS Foundation Trust or NHS Greater Glasgow and Clyde was utilized in this analysis.
