# Supplementary material for: Incidence of gout in spondyloarthritis
Source: Rheumatol Adv Pract. 2026 Mar 28;10(2):rkag039. doi: 10.1093/rap/rkag039 (PMC13070631; doi:10.1093/rap/rkag039)
Supplement: rkag039_Supplementary_Data [file rkag039_supplementary_data.doc]

Supplementary materials for:

Incidence of Gout in Spondyloarthritis

Stuart Johnston^1,2^, Benjamin Zuckerman^3^, Aashmi Sharma^4^, Uazman Alam^5^, Sizheng Steven Zhao ^4,6^

Author affiliations:

1. School of Cardiovascular and Metabolic Health, University of Glasgow, Glasgow, UK.
2. Rheumatology Physiotherapy, South Sector, NHS Greater Glasgow and Clyde, Glasgow, UK.
3. Centre for Rheumatic Diseases, King’s College London, London, UK
4. Centre for Musculoskeletal Research, Division of Musculoskeletal and Dermatological Science, School of Biological Sciences, Faculty of Biological Medicine and Health, The University of Manchester, Manchester Academic Health Science Centre, Manchester, UK
5. Department of Cardiovascular & Metabolic Medicine, Institute of Life Course and Medical Sciences, Liverpool Centre for Cardiovascular Science, University of Liverpool and Liverpool University NHS Foundation Trust
6. NIHR Manchester Biomedical Research Centre, Manchester University NHS Foundation Trust, Manchester

Correspondence to: Dr Sizheng Steven Zhao

[Sizheng.zhao@manchester.ac.uk](mailto:Sizheng.zhao@manchester.ac.uk)

Centre for Musculoskeletal Research, Division of Musculoskeletal and Dermatological Science, School of Biological Sciences, Faculty of Biological Medicine and Health, The University of Manchester, Manchester

SJ: 0009-0000-0087-1445

SZ: 0000-0002-3558-7353

Supplementary Table S1: ICD-10 codes used to define specific conditions and exclusion criteria, including corresponding ICD-10 codes.

| **Conditions** | **ICD-10 code** | **Criteria** |
| --- | --- | --- |
| Psoriatic arthritis (PsA) | L40.5 | Excluding any codes for ankylosing spondylitis (M45) |
| Cutaneous only psoriasis vulgaris (PsC) | L40.0 | Excluding PsA (L40.5) |
| Enteropathic arthritis (EA) | M07 | Plus Inflammatory bowel disease (K50 or K51) |
| Axial spondyloarthritis (AxSpA) | M45 | Excluding PsA L40.5, and M05-M07 |
| Rheumatoid arthritis (seropositive only) | M05 | Excluding M06, L40.5 and M45. |
